# Conspiracy Theories, Trust in Science, and Knowledge during the Third Wave of the COVID-19 Pandemic in Cyprus

**DOI:** 10.3390/ijerph20176710

**Published:** 2023-09-04

**Authors:** Marilena Mousoulidou, Michailina Siakalli, Andri Christodoulou, Marios Argyrides

**Affiliations:** Department of Psychology, Neapolis University Pafos, Paphos 8042, Cyprus; m.siakalli@nup.ac.cy (M.S.); andri.christodoulou@nup.ac.cy (A.C.); m.argyrides.1@nup.ac.cy (M.A.)

**Keywords:** COVID-19, pandemic, conspiracy theories, trust in science, knowledge, Cyprus

## Abstract

Conspiracy theories flourish during periods of crisis. One way to counteract the believability of conspiracy theories is trust in science and knowledge about the “perceived threat”, such as the SARS-CoV-2 virus. A total of 363 adults from Cyprus were recruited via convenience and snowball sampling methods. The data were collected via an internet-based questionnaire that examined participants’ belief in 17 conspiracy theory statements, trust in science and scientists, knowledge about the symptoms and the spread of the SARS-CoV-2 virus, phobic anxiety, hostility, somatization, and personality traits based on the Big Five. The results suggest (a) the overall belief in conspiracy theories in Cyprus is low, even though a notable percentage holds a neutral stance towards these theories; (b) trust in science and knowledge about the symptoms and the spread of the SARS-CoV-2 virus is negatively related to conspiracy theories, indicating the importance of trusting science and having knowledge as a means to counteract conspiracy theories; (c) young adults, those residing in rural areas, and those with a low education level are more likely to believe in conspiracy theories; (d) there are no significant associations between conspiracy theories and the psychological dimensions of phobic anxiety, hostility, and somatization; (e) there is difficulty in identifying specific traits related to conspiracy ideation. Public health officials could benefit from the findings when communicating information during periods of crisis.

## 1. Introduction

The SARS-CoV2 virus had been spreading across the globe since December 2019. In March 2020, the World Health Organization (WHO) declared the COVID-19 disease a pandemic [1]. Soon after, COVID-19 turned into a global crisis, and alongside the rapid spread of the virus, an extensive and rapid spread of information surrounding its origins, transmissibility, and progression was observed [2]. Reliable and credible sources, such as the WHO and the Center for Disease Control and Prevention (CDC), tried to keep the public informed using technology and social media for the dissemination of information about the virus’s symptoms, the preventive measures that needed to be implemented, and the global picture of the spread of the virus [3,4]. Following the same course, the public shared information from different sources to keep their families, friends, and community informed. However, the information shared by the public occasionally came from unsubstantiated sources, not all of which were reliable and accurate. Amongst this information were conspiracy theories, misinformation, and fake news, which resulted in more uncertainty, distorted risk perception, and mistrust of reliable sources [5,6]. Consequently, health authorities had the challenge of what the WHO would term an infodemic: “an overabundance of information, some accurate and some not, that makes it hard for people to find trustworthy sources and reliable guidance when they need it” [7] (p. 2).

When bombarded with massive amounts of information, individuals need to decide which information is accurate and which is not. The literature suggests that people frequently make decisions depending on the level of trust they display in the source of information. Trust is a complex construct influenced by many factors which may result in one person having high trust in one dimension (e.g., science) but lower in another (e.g., government) [8]. Generally, trustworthy sources are more likely to be viewed as accurate [8]. Multiple studies have shown that one specific type of trust, trust in science, plays a critical role during major public health crises [9]. Trust in science is twofold: referring to (a) trusting the scientific work of the scientists, and (b) trusting the information that originates from scientists [8]. During the COVID-19 pandemic, political decisions, such as the preventative measures that needed to be implemented, largely depended on the suggestions put forward by scientists and scientific institutions. Therefore, it is not surprising that the level of an individual’s trust in science plays a significant role in one’s decision and, eventually, one’s response concerning novel situations [9].

It has been found that the level of trust in science is related to the information people consider accurate and valid. Several studies have shown that susceptibility to conspiracy theories and misinformation is associated with low levels of trust in science, and people who score higher in trust in science are less likely to believe conspiracy theories and misinformation about COVID-19 [2,10,11,12]. This is particularly challenging for health authorities and the population since accepting conspiracy theories and misinformation as valid could influence a person’s response to the preventive measures in combating the pandemic. Studies that examined a possible association between holding a conspiracy belief and adherence to the implemented measures have shown that individuals who believe in COVID-19 conspiracy theories are more likely to have negative attitudes toward adherence to the preventive measures [12,13,14,15,16], as well as to vaccination against the virus [17]. Even though this relationship was not present in all studies [10,13], most researchers agree that conspiracy theories play an essential role in the pandemic.

Believing in conspiracy theories is a complex phenomenon, and research has identified many factors that appear to influence a person to endorse these theories. Such beliefs can be problematic during health emergencies since they can influence human behavior. Studies that examined the tendency to believe in conspiracy theories, also called conspiracy mentality [18,19,20], have shown that critical periods of uncertainty and fear give rise to conspiracy theories in an attempt to answer and understand complex, unknown, and threatening situations [21,22]. Therefore, it is unsurprising that conspiracy theories quickly grew during COVID-19.

The “profiling” of people who believe in conspiracy theories has been a matter of research interest. Some characteristics related to conspiracy mentality are younger age, male, low education, and low income [18,23]. Other research focused on the personality traits of the Big Five model (agreeableness, openness to experience, neuroticism, extraversion, and conscientiousness) [24]. The results, however, were puzzling. For example, several studies have shown that less agreeable people (agreeableness is associated with being trusting, sympathetic, and not critical) have higher conspiracy beliefs [25,26,27], whereas other studies did not [19,28]. Similarly, whereas some studies showed that the personality trait of openness to experience (associated with an active imagination and intellectual curiosity) is positively related to general conspiracist beliefs [25], other studies did not find such a link [28]. It is worth noting that mixed results were also found for the other Big Five personality traits (see [29] for a review).

The general knowledge of the symptoms and the spread of the SARS-CoV-2 virus also seems to be crucial. Examinations of the relationship between demographics (age, sex, educational level, etc.) and knowledge-score (henceforth K-score) have shown that females [30], older aged individuals [31], and individuals with a higher educational level [32] have a higher K-score. Additionally, the literature suggests that lower K-scores indicated a higher likelihood of believing conspiracy theories [31,32]. Examinations of the relationship between K-score and trust have shown that higher levels of trust also had higher K-scores [33].

The association between believing conspiracy theories and psychological well-being has also been examined in the literature. Studies have shown that conspiracy theories are related to various aspects of mental health, such as anxiety, psychological distress, depression, and life satisfaction [32,34,35]. For instance, in a systematic review, van Mulukom et al. [35] noted that COVID-19 conspiracy beliefs are associated with higher anxiety and vice versa. This area is subject to active research, and the impact of COVID-19 on other dimensions of psychological well-being, such as phobic anxiety, hostility, and somatization, remains to be established.

The main aim of the current study was to explore the prevalence of belief in conspiracy theories among citizens of Cyprus after the end of the third wave of the pandemic (July 2021). Cyprus is of great interest in the literature on conspiracy theories since it has the second highest rate of incorrectly accepting conspiracy theories in Europe [36]. In detail, a recent Special Eurobarometer that took place during April and May 2021 showed that 74% of Cyprus citizens endorsed the statement “Viruses have been produced in government laboratories to control our freedom” [36] (p. 3). This was the second highest percentage (after Bulgaria) among the European Union Member States. Additionally, Cyprus had the second highest rate of agreement (after Bulgaria) among the European Union Member States concerning the statement “The cure for cancer exists but is hidden from the public by commercial interests” [36] (p. 3). Specifically, only 27% of Cyprus citizens considered this statement false. Thus, a need to further explore the level of belief in conspiracy theories among citizens of Cyprus was deemed necessary.

Another aim of the current study was to examine the relationship between believing in conspiracy theories and trust in science. The Special Eurobarometer showed that when citizens of Cyprus are asked questions about their perceptions of scientists, they have low trust in scientists [36]. For instance, Cyprus had the highest rate (71%) of agreement with the statement “we can no longer trust scientists to tell the truth about controversial scientific and technological issues because they depend more and more on money from industry” [36] (p. 199) (see Special Eurobarometer for more details). As previously discussed, the relationship between trust in science and believing in conspiracy theories is identified in the literature. We considered it essential to examine whether this relationship is also relevant to the citizens of Cyprus.

To our knowledge, only a few studies were conducted in Cyprus that examined the relationship between believing in conspiracy theories and trust in science during the COVID-19 pandemic. One such study, conducted by Constantinou et al. [23], examined the beliefs of Cypriots and Greeks regarding various conspiracy theories circulating during the first wave of the COVID-19 pandemic. They found that individuals with stronger conspiracy beliefs had lower trust in science and adherence to public health measures. The current study was conducted in Cyprus between 23 June 2021 to 7 July 2021, a period that can be described as the end of the third pandemic wave. During this period, (a) vaccination against the virus was available nationally to people over 18 [37], and (b) strict measures started to loosen (e.g., curfew), while new measures were being implemented (i.e., mandatory frequent rapid antigen tests, the use of a “safe pass” for indoor and outdoor spaces) [38]. We consider the period of our study important since, as the uncertainty surrounding the vaccine grew, so did the conspiracy theories, dividing the public opinion about the vaccine’s effectiveness, necessity, and side effects.

The final aim of the current study was to assess the association between the acceptance of conspiracy theories and the knowledge one has about the symptoms and the spread of the SARS-CoV-2 virus. Previous research [31,32] suggests that the higher the knowledge one has about COVID-19, the lower the likelihood of believing in conspiracy theories and misinformation statements. This pattern was of interest to be examined within the population of Cyprus. For the current study, the following variables were of interest: (a) the level of belief in conspiracy theories in the population; (b) the level of trust in science in the population; (c) the K-score of individuals regarding the symptoms and the spread of the SARS-CoV-2 virus. Based on the existing literature and the purpose of the study, six hypotheses were formulated:

**Hypothesis 1 (H1).**  *There will be a significant negative association between conspiracy theories and trust in science. Based on previous literature, we expected that participants with a high level of trust in science would be less likely to rate conspiracy theories as believable.*

**Hypothesis 2 (H2).**  *There will be a significant negative relationship between K-score and conspiracy theories. In line with previous findings, we hypothesized that the higher the individual’s knowledge about the symptoms and transmissibility of the virus, the lower their belief in conspiracy theories.*

**Hypothesis 3 (H3).**  *There will be a significant positive association between individuals’ K-scores and trust in science. As previous findings suggest, we hypothesized that individuals with a higher level of trust in science would have more knowledge about the symptoms and transmissibility of the virus.*

**Hypothesis 4 (H4).**  *There will be associations between demographics and all variables of interest (conspiracy theories, trust in science, and K-score). Following the literature, we expected that (a) less educated people and those of a younger age would be more likely to rate conspiracy theories as believable, and (b) the K-score of older and more highly educated individuals would be higher.*

**Hypothesis 5 (H5).**  *There will be associations between the psychological dimensions of phobic anxiety, hostility, and somatization and all variables of interest (conspiracy theories, trust in science, and K-score). To our knowledge, no previous literature has explored these dimensions; therefore, they are of interest.*

**Hypothesis 6 (H6).**  *There will be associations between the Big Five personality traits and all variables of interest (conspiracy theories, trust in science, and K-score). The literature has produced mixed results about the association between personality traits and the variables we examined; therefore, their relationship was explored.*

## 2. Materials and Methods

### 2.1. Participants

The sample comprised 363 participants (96 males (26.4%) and 267 females (73.6%)). The vast majority (322; 88.7%) of the sample had Greek-Cypriot nationality, and 293 (80.7%) lived in an urban setting. The age of the sample ranged from 18 to 74, with 80 participants (22.04%) being young adults (18–30 years old), 240 participants (66.12%) early middle-aged adults (31–50 years old), and 43 participants (11.84%) late middle-aged/older adults (51+ years old). Concerning educational level, 39 participants (10.74%) had completed lower and/or higher secondary education, 137 (37.74%) held a bachelor’s degree, and 187 (51.52%) a master’s and/or doctorate degree.

### 2.2. Procedure

The research received the necessary ethical approval from the Cyprus National Bioethics Committee (ΕΕΒΚ ΕΠ 2021.01.150), and all research procedures were performed in accordance with their guidelines and regulations. This was a cross-sectional study aiming to recruit as many participants as possible. Invitation calls to participate in the study were posted by the authors via social media (e.g., Facebook) and social chatting apps (e.g., Viber, WhatsApp, Messenger), and were emailed to personal contacts. Interested individuals had to complete an internet-based questionnaire (created via Google Forms) that was approximately 15 min long. All participants were contacted electronically and informed about the purpose of the study. Their anonymity was assured and consent to participate in the study was ascertained prior to completing the questionnaire. Data were collected via convenience and snowball sampling methods between 23 June 2021 and 7 July 2021.

### 2.3. Measures

#### 2.3.1. Demographic Information

Data were collected on participants’ age, gender, education level, and place of residence.

#### 2.3.2. Conspiracy Theories Questionnaire (CTQ)

The CTQ [39] was employed to measure participants’ belief level in conspiracy theory statements. The CTQ is a 17-item questionnaire that was developed by the authors based on two criteria: (a) the conspiracy theory statements were prevalent in Cyprus during the period of the study, and (b) the statements were circulating on social media during the time the study was conducted. The statements were selected by searching the web for popular conspiracy theories (see, for example, [40,41]), as well as by using conspiracy theories that had been previously used in other studies (e.g., [2,12]). Participants had to rate how strongly they believed the statements on a 5-point Likert-type scale ranging from 1 (extremely unbelievable) to 5 (extremely believable).

#### 2.3.3. Trust in Science and Scientists Inventory

The Trust in Science and Scientists Inventory [42] ([43] for the Greek version) was employed to explore the participants’ level of trust in science and scientists. It contains 21 items rated on a 5-point Likert-type scale ranging from 1 (strongly disagree) to 5 (strongly agree). The items’ internal reliability (listwise deletion) was very high, with Cronbach’s alpha of 0.905 and McDonald’s omega of 0.930.

#### 2.3.4. K-Score about COVID-19

To evaluate the K-score of the participants, a 17-item questionnaire was developed. The items were designed based on WHO’s guidelines and common myths about COVID-19 at that time [44,45,46]. The questionnaire included 7 items related to the symptoms of COVID-19, such as “one severe symptom of COVID-19 is shortness of breath”, and 10 items related to how the virus is transmitted, such as “Cold weather and snow CANNOT kill the COVID-19 virus”. To minimize response bias, 7 questions were rephrased to be false. For instance, the original statement by WHO “Water or swimming does not transmit the COVID-19 virus”, was changed to “water or swimming transmits the COVID-19 virus”. Correct responses received 1 point, and incorrect ones received no points. The K-score was calculated using the percent of correct responses to all 17 items.

#### 2.3.5. Symptom Checklist-90 (SCL-90)

To measure participants’ phobic anxiety, hostility, and somatization levels, three subscales of the Symptom Checklist-90 [47] ([48] for the Greek version) were used. Specifically, the 7-item Phobic Anxiety subscale (measuring persistent irrational and disproportionate fear of the stimulus that leads to avoidance or escape behaviors); the 6-item Hostility subscale that reflects thoughts, feelings, and actions that characterize the emotional state of anger, such as aggression, irritability, rage, and indignation; and the 12-item Somatization subscale that reflects discomfort arising from perceived bodily dysfunctions. The items are self-rated on a 5-point Likert-type scale ranging from 0 (Not at All) to 4 (Extremely), specifying the level of distress experienced over the past seven days.

#### 2.3.6. Five-Item Personality Inventory (FIPI)

The Five-Item Personality Inventory (FIPI) was used [49] ([50] for the Greek version) to assess personality traits. This five-item questionnaire examines the Big Five personality traits of Openness to Experience, Conscientiousness, Extraversion, Agreeableness, and Neuroticism. Particularly, participants are asked one question per facet starting with the stem “I see myself as:” “…open to new experiences” (for Openness to Experience), “…dependable and self-disciplined” (for Conscientiousness), “…extroverted and enthusiastic” (for Extraversion), “…warm and sympathetic to others” (for Agreeableness), and “…anxious and easily upset” (for Neuroticism). All items are rated on a 5-point Likert-type scale ranging from 1 (strongly disagree) to 5 (strongly agree). While single-item questionnaires have limitations when used to measure Big Five personality traits, the scores of FIPI have adequate test-retest reliability for two weeks and adequate levels of convergent validity with other measures of the Big Five [49].

### 2.4. Statistical Analyses

All data were entered and analyzed using the Statistical Package for Social Sciences, Version 25.0 (SPSS 25, IBM Corporation, Armonk, NY, USA). The level of significance for the tests was set to 5%, and all the necessary analyses were conducted. Descriptive statistics were computed. To evaluate the prevalence of beliefs in each of the 17 conspiracy theories of CTQ ([39], see also [51]), three percentile groupings were created (1) 1st to 25th percentile represented a no-to-weak belief, (2) 26th to 75th percentile denoted a moderate belief, and (3) 76th to 100th percentile represented a strong belief. Subsequently, we conducted Pearson correlations, *t*-tests, ANOVAs, and hierarchical multiple regression to examine the variables of interest.

## 3. Results

Table 1 shows the percentages of the 363 participants to each of the 17 conspiracy theories of CTQ ([39], see also [51]) for each of the three groups. As it is clear, each statement was rated differently by the participants.

The Pearson correlation was used to explore possible associations between the variables of conspiracy theories and trust in science. The results showed a strong negative statistically significant correlation between the two variables (r = −0.772, *p* < 0.001, two-tailed). Namely, the higher the participants’ belief in conspiracy theories, the lower their trust in science level; thus, hypothesis 1 was supported (see Table 2).

The results showed a weak negative statistically significant correlation between the variables conspiracy theories and K-score (r = −0.280, *p* < 0.001, two-tailed). The higher the level of belief in conspiracy theories, the lower the value of the K-score; thus, hypothesis 2 was also supported (see Table 2).

To assess hypothesis 3, the relationship between trust in science and K-score was examined using the Pearson correlation. The analysis revealed a statistically significant positive association between trust in science and K-score (r = 0.208, *p* < 0.001, two-tailed). A higher level of trust in science was associated with a higher K-score. To explore the K-score of the participants, they were assigned to either the low K-score group (below 50% accuracy), moderate K-score group (50–84% accuracy) or the high K-score group (above 84% accuracy). The results showed that 6 participants (1.7%) had a low K-score, 265 participants (73%) had a moderate K-score, and 92 participants (25.3%) had a high K-score. Thus, this hypothesis was also supported.

We examined possible statistically significant differences between demographics and all variables of interest (conspiracy theories, trust in science, and K-score). A t-test was used to explore possible statistically significant gender differences in the variables of interest. Statistically significant differences were revealed for the variable K-score (t(361) = −2.136, *p* = 0.033, eta-squared = 0.0012) where women (M = 13.42, SD = 1.74) had higher average scores than men (M = 12.99, SD = 1.83). For the other two variables of interest, there were no statistically significant differences between men and women (*p* > 0.05). The t-test for the place of residence (urban, rural) showed statistically significant differences for all variables of interest, that is, conspiracy theories (t(361) = −2.57, *p* = 0.011, two-tailed, eta-squared = 0.018), trust in science (t(361) = 3.65, *p* < 0.01, two-tailed, eta-squared = 0.035), and K-score (t(361) = 2.907, *p* = 0.004, two-tailed, eta-squared = 0.022). For the variable conspiracy theories, higher average scores were obtained for the participants living in rural areas (M = 44.07, SD = 12.56) compared to the participants living in urban areas (M = 40.17, SD = 11.10). The opposite was found for trust in science and K-score, where higher scores were observed for the participants living in urban areas (M = 73.9, SD = 11.64 for trust in science; M = 13.45, SD = 1.7 for K-score) than those living in rural areas (M = 68.14, SD = 12.75 for trust in science; M = 12.77, SD = 1.97 for K-score). A one-way ANOVA was conducted to explore the impact of age (young adults, early middle-aged adults, and late middle-aged/older adults) for each of the three variables of interest. The analysis revealed statistically significant age differences for the variables conspiracy theories (F(2, 360) = 3.32, *p* = 0.037, eta-squared = 0.018) and trust in science (F(2, 360) = 3.26, *p* = 0.04, eta-squared = 0.018). No statistically significant differences were found for the variable K-score (*p* > 0.05). Post hoc comparisons using Tukey’s test were applied for all significant variables. For the variable conspiracy theories, differences were obtained between young adults and early middle-aged adults, with young adults having higher mean scores (M = 43.81, SD = 12.03) than early middle-aged adults (M = 40.17, SD = 11.39). No differences were obtained for the rest of the age group comparisons. For the variable trust in science, a Tukey’s post hoc test indicated statistically significant differences between young adults and late middle-aged/older adults, with late middle-aged/older adults having higher mean scores (M = 76.25, SD = 10.42) than the young adults (M = 70.51, SD = 12.3). No differences were found between the other age group comparisons. To explore the relationship between the variables of interest and the educational level, one-way ANOVA tests were conducted. Participants were divided based on their education level into three groups. Low education-level participants completed lower and/or higher secondary education; moderate education-level participants completed a bachelor’s degree; and high education-level participants completed a master’s and/or a doctorate degree. Statistically significant differences were observed for all variables of interest, i.e., conspiracy theories (F(2, 360) = 11.34, *p* < 0.001, eta-squared = 0.059), trust in science (F(2, 360) = 9.83, *p* < 0.001, eta-squared = 0.052), and K-score (F(2, 360) = 11.27, *p* < 0.001, eta-squared = 0.059). Tukey’s post hoc tests were applied. Namely, for the variable conspiracy theories, differences were obtained for low education-level and high education-level groups (M = 43.62, SD = 11.26 vs. M = 38.21, SD = 10.69) and for moderate education-level and high education-level groups (M = 43.85, SD = 11.78 vs. M = 38.21, SD = 10.69). In both pairwise comparisons, the high education-level group had lower scores than the other two groups. For the variable trust in science, statistically significant differences were observed between low education-level and high education-level groups, with the low education-level group having a lower mean level of trust in science (M = 69.15, SD = 12.25) than the high education-level group (M = 75.43, SD = 11.26). Additionally, statistically significant differences were found between the moderate education-level group and the high education-level group, with high education-level participants having a higher level of trust in science (M = 75.43, SD = 11.26) compared to moderate education-level participants (M = 70.22, SD = 12.33). For the variable K-score, there were differences between the low education-level group and the moderate education-level group and between the low education-level group and the high education-level group. Specifically, on both pairwise comparisons, the low education-level group had lower average K-scores (M = 12.18, SD = 2.21) than both the moderate education-level (M = 13.25, SD = 1.6) and the high education-level (M = 13.61, SD = 1.7) groups. Thus, hypothesis 4 was partially supported.

Concerning hypothesis 5, each of the Big Five personality traits (Openness to Experience, Conscientiousness, Extraversion, Agreeableness, and Neuroticism) was tested for statistically significant correlation with each of the three variables of interest. There was a statistically significant positive correlation between conspiracy theories and Agreeableness (r = 0.116, *p* = 0.027, two-tailed). In addition, there was a significant positive correlation between the K-score and Conscientiousness (r = 0.178, *p* < 0.001, two-tailed), thus partially supporting hypothesis 5 (see Table 2).

Concerning hypothesis 6, we examined possible correlations between the three subscales of the SCL-90 (Phobic Anxiety, Hostility, and Somatization) and the variables of interest. There was a statistically significant negative association between trust in science and Hostility (r = −0.169, *p* < 0.01, two-tailed), and between trust in science and Somatization (r = −0.172, *p* < 0.01, two-tailed), that is, the higher the trust in science, the lower the level of Hostility and Somatization. A significant negative association was also found between K-score and Hostility r = −0.120, *p* < 0.05, two-tailed), with individuals with a higher K-score having a lower level of Hostility. The results showed no associations between the variable conspiracy theories and any of the three subscales of the SCL-90 (*p* > 0.05). Thus, hypothesis 6 was also partially supported (see Table 3).

Hierarchical multiple regression was used to assess the ability of two control measures (K-score and trust in science) in order to predict the level of belief in conspiracy theories after controlling for the influence of the demographical variables (gender, place of residence, age, and educational level). The highest VIF for the aforementioned variables was 1.121, and the highest tolerance was 0.996, implying no violation of the multicollinearity assumption. In addition, the assumptions of normality, linearity, independence, and homoscedasticity were approximately satisfied. The first model included the demographical variables and was significant (F(4, 358) = 6.174, *p* < 0.01), explaining 7% of the variance in conspiracy beliefs ($R^{2}=0.07)$. The second model additionally included the K-score variable (F(5, 357) = 9.745, *p* < 0.01), and the total variance explained by the model as a whole was 12% ($R^{2}=0.12)$ of the variance. The K-score variable explained an additional 5% of the variance in conspiracy beliefs and was significant (*p* < 0.01) after controlling for the demographic variables (ΔF = 20.143, $\Delta R^{2}$ = 0.05, *p* < 0.01). The final model in which the variable trust in science was added was significant (F(6, 356) = 70.195, *p* < 0.01) and accounted for 54.2% of the total variance in conspiracy beliefs. The final model with the addition of the variable trust in science showed a significant improvement from the second model (ΔF = 327.839, $\Delta R^{2}$ = 0.422, *p* < 0.01), with statistically significant variables being the K-score (*p* < 0.01) and the trust in science (*p* < 0.01), which were negatively related with the level of belief in conspiracy theories (see Table 4).

## 4. Discussion

The first aim of our study was to explore the prevalence of belief in conspiracy theories among citizens of Cyprus after the end of the third wave of the pandemic in July 2021. Participants’ responses to the 17 conspiracy theories were grouped into no-to-weak belief, neutral belief, and moderate-to-strong belief. Interestingly, more than half of our participants had a no-to-weak belief regarding 11 of the 17 statements (percentages ranging between 50.1% to 86.2%), with the conspiracy theory claiming that the vaccine includes a tracking device being the one least supported. The current results do not reflect the prevalence of strong beliefs (20–50%) observed in another study that was conducted in Cyprus [23]. Note, however, that the current study was conducted during a different period, which might have influenced our findings. Furthermore, our results do not reflect a strong believability score in conspiracy theories for the population of Cyprus, as has been suggested by the Special Eurobarometer [36]. This could be possibly due to the different sampling methods and/or to the questions that explored the believability of conspiracy theories used in the current study. Our results show that participants had a moderate-to-strong belief only concerning the conspiracy theory statement “COVID-19 escaped from a lab in China” (54.8%). Importantly, what we believe reflects our results is that uncertainty prevailed during the period that this research was conducted. We found that one-third of the participants took a neutral stance toward eight statements (percentages ranging between 30% to 40.5%), with the conspiracy theory statement “the COVID-19 vaccine can affect women’s fertility” having the most neutral responses (40.5%). In line with this argument is the finding that participants’ opinions were divided concerning the statement “researchers rushed the development of the COVID-19 vaccine, so its effectiveness and safety cannot be trusted” (35.5% for no-to-weak belief, 33.1% for neutral, and 31.4% for strong belief). Worth noting is the fact that, in a different survey, we directly examined the relationship between belief in vaccination-related conspiracy theories and vaccine decisiveness of the population in Cyprus, and our results showed that belief in conspiracy theories predicted vaccination decisiveness [51]. Moreover, Mousoulidou et al. [51] divided the sample into vaccinated and non-vaccinated individuals and examined their belief in ten vaccine-related items from the CTQ [39]. In terms of how individuals rated the conspiracy theory statements, Mousoulidou et al. [51] found that the neutral response rates for non-vaccinated individuals ranged from 21.7% to 39.2%, whereas for vaccinated individuals, the neutral response rates ranged from 3.3% to 42.8% (see [51] for more details of this research). Having a neutral response is very important since these individuals will eventually need to take a stance. This study occurred when the vaccination against the virus was already available to citizens of Cyprus over the age of 18. Not having a clear view of the vaccine’s effectiveness and yet having to make the critical decision of getting vaccinated or not can be problematic. This finding might indicate that, during that time, the information provided by public health officials was insufficient for the citizens of Cyprus to reject these statements.

Further support for this view is the finding that 73% of our participants had a moderate K-score. Interestingly, even though the current research was conducted more than a year after COVID-19 first appeared, the participants had many gaps in knowledge concerning the symptoms and transmission of the virus. Though the new information that is continuously evolving justifies some gaps in knowledge, the current results pinpoint the importance of the population’s awareness about public health emergencies. Our results pinpoint the necessity of public health officials to find ways to increase the population’s knowledge during periods of crisis. This knowledge will help individuals protect themselves from the virus and minimize the possibility of individuals’ endorsement of conspiracy theories. For instance, a number of studies examining vaccine-related knowledge found that the better the understanding of vaccination, the more likely people are to choose to be vaccinated against the SARS-CoV-2 virus [52,53,54,55,56,57].

Our findings also showed a significant negative correlation between conspiracy theories and trust in science. As we hypothesized, and supporting previous findings [2,10,11,12], our results suggest that those who believe in conspiracy theories are less likely to trust science and scientists. We also found that knowledge about the symptoms and the spread of the SARS-CoV-2 virus is negatively related to belief in conspiracy theories. Thus, trust in science and K-score play a significant role in conspiracy theories, with higher scores in these variables possibly acting as protective factors against belief in conspiracy theories.

When examining how the demographics relate to conspiracy theories, results suggest that young adults, individuals who reside in rural areas, and individuals of low education level are more likely to believe in conspiracy theories. This finding is in concordance with the literature [18,23]. The examination of possible associations between demographics and trust in science showed that individuals older than 51 and individuals with a high education level had higher trust in science. Moreover, our results showed significant statistical differences between knowledge about the symptoms and the spread of the virus and gender. In accordance with previous studies, women were found to have a higher K-score than men [30]. K-scores also significantly differed by educational level, with low education-level individuals having a lower K-score when compared to both moderate education-level individuals, and high education-level individuals. This replicates the findings from previous research [32]. Taken together, the demographics suggest that education is an important variable relating to the three variables we examined. High education-level individuals are less likely to believe in conspiracy theories, more likely to trust science, and more likely to have higher knowledge about the symptoms and the spread of the virus.

Further investigations of the overall belief in conspiracy theories showed that believability was impacted by trust in science and K-score. This appears to suggest that high trust in science and scientists and high knowledge about the symptoms and the spread of the SARS-CoV-2 virus influenced the participants’ belief in conspiracy theories. It is important, therefore, for public health officials to find ways to increase the trust in science and the K-score level of individuals. This will not only “protect” individuals from believing in conspiracy theories but will also increase their knowledge on how to successfully combat the pandemic.

The examination of a possible association between conspiracy theories and the psychological dimensions of phobic anxiety, hostility, and somatization did not show any significant differences. Since these dimensions were not previously studied concerning conspiracy theories, trust in science, and K-score during the COVID-19 era, more research is encouraged in this direction.

Lastly, the results regarding the relationship between the Big Five personality traits and belief in conspiracy theories differed from those reported in the literature. Previous research suggested a negative association between agreeableness and conspiracy ideation [25,27], whereas our results showed that highly agreeable individuals were more likely to endorse conspiracy beliefs. Further examinations must be employed for a clearer understanding of this relationship. Our results support the difficulty in identifying specific traits related to conspiracy ideation and are in line with the existing literature that produced mixed findings [19,28].

The current study provides an important contribution to the literature, as it shows the prevalence of belief in conspiracy theories among citizens of Cyprus, as well as how trusting science and knowledge influence this belief. Nevertheless, it is limited in the following ways. Firstly, since more than half of our participants had a master’s and/or a doctorate degree, generalizations should be avoided. Additionally, there was an imbalance regarding gender and place of residence which may have affected our findings. In addition, the period in which the current study occurred may have influenced our findings. Lastly, the use of self-report methodology and convenience sampling could impact the generalizability of the current findings and should always be viewed with caution. It is also important to note that other factors found in the literature related to conspiracy theories that were not examined in the current research may have had an impact on our results. This includes, but is not limited to, analytic reasoning [58,59,60,61], teleological thinking [62], scientific reasoning [63], and religiosity [10,64,65].

## 5. Conclusions

In conclusion, the current study examined the belief in conspiracy theories by citizens of Cyprus and how it relates to trust in science and knowledge about the symptoms and the spread of the SARS-CoV-2 virus. Our results suggest that conspiracy theories do not prevail among Cyprus citizens; instead, we found that a respectable percentage of our participants did not have a clear stance toward conspiracy theories, responding with neutrality to eight conspiracy theories. Notably, the current research demonstrates the significance of trust in science and knowledge as a means to counteract conspiracy theories. Our findings suggest that individuals who believe in conspiracy theories are more likely to have a lower level of trust in science and less knowledge about the symptoms and the spread of the SARS-CoV-2 virus. The current findings have critical implications for public health officials by pinpointing the importance of finding ways to increase trust in science to counterbalance the effect of misinformation and conspiracy theories. Well-organized campaigns investing in science communication could increase awareness of the importance of the work of the scientific community to healthcare professionals and the general population. Additionally, the dissemination of evidence-based information could promote a scientific culture that will be a very beneficial resource in facing future crises.

## Figures and Tables

**Table 1:** Mean responses and percentage of no-to-weak, neutral, and moderate-to-strong belief endorsement for each conspiracy theory statement.

How Strongly Do You Believe the Following Statements:	MEAN (SD)	SEM	% No-to-Weak Belief	% Neutral Belief	% Moderate-to-Strong Belief
The emergence of the novel COVID-19 pandemic is related to the installation of 5G cellphone networks.	1.68 (0.968)	0.051	78.2	16.8	5
2. Bill Gates is involved in the spread of COVID-19 in order to expand his vaccination programs.	2.03 (1.165)	0.061	64.2	24.5	11.3
3. COVID-19 was created as a biological weapon by China, the United States, or some other country.	2.83 (1.230)	0.065	36.9	33.9	29.2
4. The COVID-19 virus originated in animals (like bats) and spread to humans.	3.19 (1.048)	0.055	21.8	36.4	41.9
5. COVID-19 escaped from a lab in China.	3.49 (0.938)	0.049	11.6	33.6	54.8
6. COVID-19 is an intentional population control scheme.	2.73 (1.289)	0.068	41	30.3	28.7
7. COVID-19 does not actually exist but is a plot to take away our personal freedom.	1.80 (1.039)	0.055	76.3	16.8	6.9
8. Conventional drugs to fight the COVID-19 virus do not cure it but are a plot by big pharmaceutical companies. There are alternative medicine available (such as vitamins and herbs) that can cure or even prevent the COVID-19 virus.	2.40 (1.197)	0.063	51.8	30	18.2
9. COVID death rates are inflated, and therefore there is no reason for lockdown regulations or other social distancing measures.	2.49 (1.215)	0.064	50.1	30.6	19.3
10. COVID-19 is no more dangerous than the flu, but the risks have been exaggerated as a way to restrict personal freedom.	2.48 (1.233)	0.065	53.5	24.2	22.3
11. The mRNA technology used by some of the vaccines alters (can alter) our DNA.	2.41 (1.144)	0.060	52.6	30.6	16.8
12. The COVID-19 vaccine can affect women’s fertility.	2.85 (1.075)	0.056	34.7	40.5	24.8
13. You can get COVID-19 from the vaccine.	2.09 (1.151)	0.06	65.6	20.4	14
14. People who received the COVID-19 vaccine no longer need to wear a mask or take any precautions against the virus	2.05 (1.084)	0.057	69.7	19	11.3
15. Researchers rushed the development of the COVID-19 vaccine, so its effectiveness and safety cannot be trusted.	2.94 (1.135)	0.060	35.5	33.1	31.4
16. The COVID-19 vaccine includes a tracking device or microchips.	1.53 (0.887)	0.047	86.2	9.4	4.4
17. People that have already been diagnosed with COVID-19 do not need to receive the vaccine.	1.95 (1.186)	0.062	71.4	16.5	12.1

Note: Mean on Likert scale 1–5, where 1 = “Extremely Unbelievable” to 5 = “Extremely Believable”; SEM = standard error of the mean; % no-to-weak belief = percentage of sample in 1st to 25th percentile; neutral belief = 26th to 75th percentile; and moderate-to-strong Belief = 76th to 100th percentile.

**Table 2:** Correlations among the Big Five personality traits and all the variables of interest.

Variables	1	2	3	4	5	6	7	8
Openness to Experience	------							
2. Conscientiousness	0.427 **	-------						
3. Extraversion	0.397 **	0.322 **	-------					
4. Agreeableness	0.396 **	0.489 **	0.452 **	-------				
5. Neuroticism	0.045	0.079	0.165 **	0.107 *	-------			
6. Trust in Science	0.003	0.025	0.053	0.033	−0.051	-------		
7. Conspiracy Theories	−0.004	−0.046	0.025	0.116 *	0.081	−0.722 **	------	
8. K-score	0.078	0.178 **	0.057	0.070	−0.013	0.208 **	−0.280 **	------

Note. Total n = 363, * *p* < 0.05, ** *p* < 0.01.

**Table 3:** Correlations among SCL-90 subscales and all the variables of interest.

Variables	1	2	3	4	5	6
Phobic Anxiety	------					
2. Hostility	0.512 **	-------				
3. Somatization	0.413 **	0.504 **	-------			
4. Trust in Science	−0.085	−0.169 **	−0.172 **	------		
5. Conspiracy Theories	0.001	0.080	0.096	−0.722 **	------	
6. K-score	−0.068	−0.120 *	−0.056	0.208 **	−0.280 **	------

Note. Total n = 363, * *p* < 0.05, ** *p* < 0.01.

**Table 4:** Results of hierarchical regression analysis predicting the level of belief in conspiracy theories.

Model	B	S.E.	*β*	*t*	$R^{2}$	$\Delta R^{2}$	ΔF
Step 1					0.07	0.07	6.714
Constant **	49.721	4.481		11.096			
Gender	−0.155	1.332	−0.006	−0.117			
Age	−1.811	1.055	−0.090	−1.718			
Educational Level **	−3.563	0.867	−0.210	−4.108			
Place of Residence	2.924	1.531	0.101	1.910			
Step 2					0.12	0.05	20.143
Constant **	67.723	5.910		11.460			
Gender	0.611	1.308	0.023	0.467			
Age	−1.825	1.027	−0.091	−1.777			
Educational Level *	−2.677	0.867	−0.158	−3.087			
Place of Residence	1.926	1.507	0.066	1.278			
K-score **	−1.520	0.336	−0.235	−4.518			
Step 3					0.542	0.422	327.839
Constant **	103.747	4.711		22.024			
Gender	−0.173	0.946	−0.007	−0.183			
Age	−0.605	0.745	−0.030	−0.811			
Educational Level	−0.693	0.636	−0.041	−1.089			
Place of Residence	−0.710	1.099	−0.024	−0.646			
K-score **	−0.843	0.246	−0.130	−3.426			
Trust in Science **	−0.654	0.036	−0.687	−18.106			

Note. * *p* < 0.01, ** *p* < 0.001.

## Data Availability

Data will be available upon reasonable request from the corresponding author.
